# Efficiency rating of SG Diagnostics COVID-19 antigen rapid test kit

**DOI:** 10.2217/fvl-2021-0210

**Published:** 2023-04-17

**Authors:** Francesco Chirico, Murat Yıldırım, Tomasz Dzieciatkowski, Marek Dabrowski, Marek Malysz, Marcin Madziala, Milosz Jaroslaw Jaguszewski, Karol Bielski, Gabriella Nucera, Krzysztof J Filipiak, Lukasz Szarpak

**Affiliations:** ^1^Post-graduate School of Occupational Health, Università Cattolica del Sacro Cuore, Rome, Italy; ^2^Health Service Department, Italian State Police, Ministry of the Interior, Milan, Italy; ^3^Department of Psychology, Faculty of Science & Letters, Agri Ibrahim Cecen University, Ağrı, Turkey; ^4^Chair & Department of Medical Microbiology, Medical University of Warsaw, Warsaw, Poland; ^5^Research Unit, Polish Society of Disaster Medicine, Warsaw, Poland; ^6^Chair & Department of Medical Education, Poznan University of Medical Sciences, Poznan, Poland; ^7^Institute of Outcomes Research, Polonia University, Czestochowa, Poland; ^8^1st Department of Cardiology, Medical University of Gdansk, Gdansk, Poland; ^9^Emergency Medical Service, Warsaw, Poland; ^10^Department of Emergency, Fatebenefratelli Hospital, ASST Fatebenefratelli & Sacco, Milan, Italy; ^11^Institute of Outcomes Research, Maria Sklodowska-Curie Medical Academy, Warsaw, Poland; ^12^Research Unit, Maria Sklodowska-Curie Bialystok Oncology Center, Bialystok, Poland

**Keywords:** antigen rapid test, coronavirus disease, COVID-19, diagnostic, RT-qPCR

## Abstract

**Aim:** Rapid detection is crucial in complementing vaccination to reduce transmission of SARS-CoV-2. **Materials & methods:** Nasopharyngeal swabs (n = 213) and oropharyngeal swabs (n = 98) were tested. with the antigen rapid test kit. **Results:** Overall sensitivity (97.96%), specificity (100.00%) and coincidence rate (98.71%) were high, which translated into a positive predictive value of 100.00% and a negative predictive value of 96.64%. **Conclusion:** Antigen rapid tests have a great potential for screening in different settings to deliver results with high sensitivity and specificity.

## Plain language summary

This study evaluated SG Diagnostics COVID-19 antigen rapid test kit. The overall sensitivity, specificity and coincidence rate were found very high with SG Diagnostics COVID-19 antigen rapid test kit performing better.

## Tweetable abstract

Antigen rapid test kit performed well for sensitivity (≥97%) and specificity (100%).

The novel coronavirus SARS-COV-2 is the triggering pathogen for the COVID-19 pandemic that has spread to 219 countries across the globe [1]. People infected with the virus suffer from mild to severe symptoms of respiratory illness and recover without particular treatment [2]. The symptoms of COVID-19 may be varied and depend on the virus variant, but the most common are fever (43–80%), headaches (13–70%), loss of smell (70%), cough (50–63%) and fatigue (31–63%). Elderly and those with pre-existing physical health problems (e.g., diabetes, cardiovascular disease, immunosuppressive treatment, chronic respiratory disease and cancer) tend to develop serious symptoms of the disease such as shortness of breath or difficulty breathing, loss of speech or movement and chest pain [2,3]. It usually takes about 5–6 days for people to be infected with the virus for symptoms to occur. However, it may take up to 14 days for some people [4,5]. Currently, many countries have experienced multiple waves of COVID-19 and vaccination is not happening fast enough to prevent/reduce transmission of the virus.

Testing for SARS-COV-2 (e.g., RT-qPCR or rapid antigen test) has been identified to be one of the approaches that complement the vaccination approach to prevent/reduce transmission [6–8]. Reverse transcriptase-polymerase chain reaction, especially in a quantitative polymerase chain reaction (RT-qPCR), is the gold standard for the diagnosis of COVID-19 infection because of its high sensitivity and specificity, as compared with rapid antigen test [9]. However, RT-qPCR has several disadvantages that limit the effectiveness of isolation and contact tracing strategies; these disadvantages include [10] the need to carry out the testing in a laboratory by well-trained personnel [11], global reagent shortage and [12] long turnaround time (may take up to 48 h). In contrast, rapid antigen test [10] can be performed at the point-of-care or at home (i.e., self-testing) [11], is inexpensive and [10,13] the turnaround time is much shorter (within minutes) and hence, rapid antigen test can greatly help to speed up detection and isolation of new cases [14].

It is also worth noting that a positive result indicates SARS-CoV-2 infection, but when the result is undetermined, it does not exclude, nor does it confirm infection [15]. In this case, it is recommended to test another sample, collected after 24–48 h [13]. Also, a single negative result does not rule out infection and should not be considered the only diagnostic criterion, especially in cases where the clinical picture suggests SARS-CoV-2 infection or the patient had close contact, without personal protective equipment, with a confirmed case of COVID-19, regardless of type and severity the clinical symptoms presented [16]. Rapid antigen tests may give positive results in the period when the amount of virus in the respiratory tract is greatest, in other words, from 1 to 3 days before the onset of symptoms up to 5–7 days after the onset of symptoms, the risk of obtaining false-negative results is greater [17]. A systematic review and meta-analysis study showed that the rapid antigen test has a varying sensitivity (71.2% [95% CI: 68.2–74.0%]) and specificity (98.9% [95% CI: 98.6–99.1%]) [18]. Sufficient performance of commercially available rapid antigen tests has been reported for public health and clinical practice in terms of analytical specificity and sensitivity [19].

In this study, we aimed to assess the performance of the SG Diagnostics COVID-19 antigen rapid test kit (hereafter referred to as ‘antigen rapid test’) with the 311 clinical specimens. This study was carried out at four testing sites in Poland. A second clinical specimen (either nasopharyngeal swab or oropharyngeal swab) was directly tested with the antigen rapid test kit on site and results were compared with the ‘gold standard’ RT-qPCR that was carried out with the first clinical specimen of the same specimen type. The results for both tests were compiled and were used to compute sensitivity, specificity, positive predictive value, negative predictive value and total coincidence rate to evaluate the performance Antigen Rapid Test.

## Materials & methods

### Testing site & participant recruitment

The study was carried out at four testing sites in Poland, and they were: an emergency medical service in Warsaw; an emergency medical service in Poznan; and a testing point in Skierniewice. The eligibility criterion for a free-of-charge test was to have either symptoms or close contact with a confirmed COVID-19 infected individual. All people over 18 years of age at the entrance of the testing site were contacted to be included in the study. Volunteers were enrolled and directed to one of the dedicated testing sites for the Ag RDT. The study was conducted for 5 days in the 1st week of January during the COVID-19 pandemic in 2021.

### Specimen collection & testing

Swabbing was carried out by healthcare professionals (i.e., nurses, physicians, paramedics and medical students) that were dressed in full personal protective equipment (PPE). Sterile swabs were used to collect upper respiratory specimens at the same time. For the nasopharyngeal swab, a mini-tip swab with a flexible shaft was slowly and gently inserted through the nostril until the experience of resistance. Then, the swab was gently rubbed and rolled. In order to absorb secretions, the swab was left in place for several seconds. While rotating the swap, it was slowly removed. The same swab was used to collect specimens from both sides. In case there was blockage from one nostril, the other nostril was used to collect the specimens. Afterward, the swap was placed into the transport tube. The volume and manufacturer of the transport medium were respectively 500 ul and Nanjing Vazyme Biotech Co. For the oropharyngeal swab, the swab was inserted into the tonsillar and posterior pharynx areas. The swab was then rubbed over the posterior oropharynx and tonsillar pillars without touching the teeth, gums and tongue. Again, the swap was placed into the transport tube. A standard method for SARS-CoV-2 testing is RT-qPCR which was carried out as usual, in parallel with the antigen rapid test kit. All participants received sufficient instructions regarding the aims of the study, their rights to withdraw from the study at any time point without giving any reasons, and anonymity and confidentiality of responses. Volunteers provided written informed consent. All testing was conducted by healthcare professionals. Before starting the study, ethical approval was obtained for the protocol of this study from the Institutional Review Board of the last author’s institution.

The first clinical specimen (either nasopharyngeal swab or oropharyngeal swab, but not both) was taken and placed directly in universal transport media for shipping to the diagnostic laboratory for RT-qPCR testing. To have more reliable and quantitative real-time data, multiple RT-qPCR detection kits were used [1]; BioFire Respiratory Panel by BioFire Diagnostics [2], Cepheid GeneXpert by CEPHEID [3], AmpliTest SARS-CoV-2 by AMPLICON [4], Path-2019-nCoV-std by Primerdesign [5] and Z-Path-COVID-19-CE by Primerdesign.

The second clinical specimen that was of the same specimen type as the first clinical specimen was taken for evaluating the antigen rapid test kit by directly comparing the RT-qPCR detection kit result with the antigen rapid test kit result. Testing with the antigen rapid test kit was done onsite by trained staff dressed in full personal protective equipment. Samples for the antigen rapid test kit were processed within 10 min for testing. The result was read after 10 min but not beyond 15 min after testing initiation. When the results were ambiguous, a readout was performed independently by two persons. The test results were recorded with paper and pen and were keyed into Microsoft Excel Spreadsheet at the end of the day by the primary researchers of this study. A quality check of data captured in the electronic sheets was also performed to reduce the input error. Both the swab and used Antigen Rapid Test Kit were discarded as biohazard waste according to legislated regulatory requirements.

### Data analysis

Data from the RT-qPCR detection kit and antigen rapid test kit was merged using Microsoft Excel Spreadsheet. Sensitivity, specificity, positive predictive value, negative predictive value and total coincidence rate of the antigen rapid test kit were calculated in relation to the RT-qPCR results based on the formula shown in Table 1.

**Table 1.** Formula for calculating sensitivity, specificity, positive predictive value, negative predictive value and total coincidence rate of antigen rapid test kit.

		+	−
Antigen rapid test results	+	True positive (A)	False positive (B)
	−	False negative (C)	True negative (D)

Sensitivity: A/(A + C) × 100%.

Specificity: D/(B + D) × 100%.

Positive predictive value: A/(A + B) × 100%.

Negative predictive value: D/(C + D) × 100%.

Total coincide rate: (A + D)/(A + B + C + D) × 100%.

## Results

### Overall performance of rapid antigen test kit

Among the 311 clinical specimens, 192 and 115 of them were reported to be positive and negative, respectively, by both the RT-qPCR kit and rapid antigen test kit (Table 2). For the remaining clinical specimens, four were reported to be positive only by RT-qPCR kit and none was reported to be positive only by Rapid Antigen Test Kit.

**Table 2.** Comparison of reverse transcriptase-quantitative PCR and test kit results of all clinical specimens.

Method		RT-PCR		Total
		Positive	Negative	
SG Diagnostics COVID-19 antigen rapid test kit	Positive	192	0	192
	Negative	4	115	119
Total		196	115	311

The overall sensitivity and specificity were 97.96% (95% CI: 94.86–99.44%) and 100.00% (95% CI: 96.84–100.00%), respectively, which translated into a positive predictive value of 100.00% and a negative predictive value of 96.64% (95% CI: 91.60–98.70%). The overall total coincidence rate was 98.71% (95% CI: 96.74–99.65%).

### Performance of rapid antigen test kit with nasopharyngeal swab specimens

Among the 213 nasopharyngeal swab specimens that were collected, 150 and 60 of them were reported to be positive and negative, respectively, by both the RT-qPCR kit and Rapid Antigen Test Kit (Table 3). For the remaining clinical specimens, three were reported to be positive only by RT-qPCR kit and none was reported to be positive only by rapid antigen test kit.

**Table 3.** Comparison of reverse transcriptase-quantitative PCR and test kit results of nasopharyngeal swabs.

Method		RT-PCR		Total
		Positive	Negative	
SG Diagnostics COVID-19 antigen rapid test kit	Positive	150	0	150
	Negative	3	60	63
Total		153	60	233

The overall sensitivity and specificity were 98.04% (95% Cl: 94.38–99.59%) and 100.00% (95% Cl: 94.04–100.00%), respectively, which translated into a positive predictive value of 100.00% and a negative predictive value of 95.24% (95% CI: 86.71–98.40%). The overall total coincidence rate was 98.59% (95% Cl: 95.94–99.71%).

### Performance of rapid antigen test kit with oropharyngeal swab specimens

Among the 98 oropharyngeal swab specimens that were collected from symptomatic and asymptomatic participants, 42 and 55 of them were reported to be positive and negative, respectively, by both the RT-qPCR kit and Rapid Antigen Test Kit (Table 4). For the remaining clinical specimens, one was reported to be positive only by RT-qPCR kit and none was reported to be positive only by Rapid Antigen Test Kit.

**Table 4.** Comparison of reverse transcriptase-quantitative PCR and test kit results of oropharyngeal swabs.

Method		RT-PCR		Total
		Positive	Negative	
SG Diagnostics COVID-19 antigen rapid test kit	Positive	42	0	42
	Negative	1	55	56
Total		13	55	98

The overall sensitivity and specificity were 97.67% (95% CI: 87.71–99.94%) and 100.00% (95% CI: 93.51–100.00%), respectively, which translated into a positive predictive value of 100.00% and a negative predictive value of 98.21% (95% CI: 88.80–99.74%). The overall total coincidence rate was 98.98% (95% CI: 94.45–99.97%).

## Discussion

In this study, the performance of the SG Diagnostics COVID-19 antigen rapid test kit (Colloidal Gold-based) was evaluated with 311 clinical specimens. Compared with the ‘gold standard’ RT-qPCR, the antigen rapid test kit demonstrated an overall sensitivity of 97.96% (95% CI: 94.86–99.44%), specificity of 100.00% (95% CI: 96.84–100.00%) and total coincidence rate of 98.71% (95% CI: 96.74–99.65%), regardless of specimen types. For the different specimen types, the sensitivity, specificity and total coincidence rate were 98.04% (95% Cl: 94.38–99.59%), 100.00% (95% Cl: 94.04–100.00%) and 98.59% (95% Cl: 95.94–99.71%) for a nasopharyngeal swab and 97.67% (95% CI: 87.71–99.94%), 100.00% (95% CI: 93.51–100.00%) and 98.98% (95% CI: 94.45–99.97%) for an oropharyngeal swab. It should be remembered that the sensitivity of molecular tests depends on the type of biological material tested. The best results are obtained with samples of the throat and nose swabs taken simultaneously (97%) and nasopharyngeal swabs (92.2%). Single oropharyngeal swabs are a much worse clinical material, where the detection sensitivity takes down to 84%. In addition, often the results of molecular tests for SARS-CoV-2 may be falsified by the presence of reaction inhibitors and/or inappropriate transport of clinical samples to certified laboratories. WHO recommends rapid antigen tests to meet the minimum performance requirements of ≥80% sensitivity and ≥97% specificity in detecting COVID-19 positive and negative individuals, respectively.

## Conclusion

Compared with the ‘gold standard’ RT-qPCR, SG Diagnostics COVID-19 antigen rapid test kit (Colloidal Gold-Based) has performed well in terms of sensitivity and specificity (≥97% sensitivity and 100% specificity). Although having lower sensitivity and specificity, tests to detect SARS-CoV-2 antigens may perform better [18,19], especially when rapid test results are needed. However, it is not recommended to use rapid antigen tests for screening (e.g., in the analysis of virus prevalence in the general population) before giving medical advice [20]. Given the advantage of the COVID-19 antigen rapid test to deliver the results quickly (within 15 min) and with the high sensitivity and specificity as shown in this study, COVID-19 antigen rapid tests have a great potential for screening both in the hospital setting as well as for the general population (self-test).

**Summary points**

- SG Diagnostics COVID-19 antigen rapid test is viewed as an important diagnostic tool to deal with the spread of SARS-CoV-2.
- There is a growing body of literature assessing the performance of SG Diagnostics COVID-19 antigen rapid test due to their increasing use in the market.
- There is an urgent need to provide evidence about their utility and performance in order to inform decision-makers.
- SG Diagnostics COVID-19 antigen rapid test kit was assessed at four testing sites on nasopharyngeal swabs and oropharyngeal swabs in Poland.
- Compared with the ‘gold standard’ RT-qPCR, the antigen rapid test kit indicated an overall sensitivity of 97.96% (95% CI: 94.86–99.44%), specificity of 100.00% (95% CI: 96.84–100.00%) and total coincidence rate of 98.71% (95% CI: 96.74–99.65%) despite specimen types.
- The sensitivity, specificity, and total coincidence rate for the different specimen types were respectively 98.04% (95% Cl: 94.38–99.59%), 100.00% (95% Cl: 94.04–100.00%) and 98.59% (95% Cl: 95.94–99.71%) for a nasopharyngeal swab and 97.67% (95% CI: 87.71–99.94%), 100.00% (95% CI: 93.51–100.00%) and 98.98% (95% CI: 94.45–99.97%) for an oropharyngeal swab.
- The COVID-19 antigen rapid test was helpful to deliver the results within 15 min with high sensitivity and specificity. Therefore, they have a great potential for screening in the early phase of disease both in the hospital setting and in the general population (self-test).
- Further clinical accuracy research would strengthen test comparisons.
